# A visualization tool for eye tracking time series analysis supporting teachers and psychologists

**DOI:** 10.1038/s41598-026-56941-4

**Published:** 2026-06-18

**Authors:** Abdul Rehman, Ilona Heldal, Jerry Chun-Wei Lin

**Affiliations:** 1 Department of Computer Science, Electrical Engineering and Mathematical Sciences, Western Norway University of Applied Sciences, Bergen, Norway; 2 Department of Distributed Systems and IT Devices, Silesian University of Technology, Gliwice, Poland

**Keywords:** Eye-tracking technology, Visual cognition, Time series analysis, Educational technology, Visual attention, Learning analytics, Behavior analysis, Mathematics and computing, Neuroscience, Psychology, Psychology

## Abstract

Eye Tracking (ET) can help improve understanding of visual attention in computer-supported interactive environments. In attention tasks, distinguishing between relevant target objects and distractors is crucial for effective performance, yet the underlying gaze patterns that drive successful task completion remain incompletely understood. Traditional gaze analyses provide limited insight into the temporal dynamics of attention allocation and the relationship between gaze behavior and task performance. When applied to complex visual search scenarios, current gaze analysis methods face several limitations, including the isolation of measurements in dynamic environments, visual stability, search efficiency, and the task-solving processes involved. This paper proposes an analysis tool, *VisiTrail*, that considers time-series eye-tracking data on task performance and gaze measures; temporal pattern analysis that reveals how attention evolves throughout task performance; object-click sequence tracking that directly links visual attention to user actions; and performance metrics that quantify both accuracy and efficiency of right actions. The proposed analysis is applied to data collected from the Mushroom Hunter serious game, a multilevel visual search task in which participants identify target mushrooms among distractors of increasing complexity across three difficulty levels. This tool focuses on two scenarios: subject-specific analysis and general analysis across all subjects from which a project collected data from Romania and Portugal. Subject-specific analysis uses only one participant’s data and yields two types of results: a first-level overall analysis that provides detailed insights and a multilevel analysis that compares performance across all three levels, where Level 1 presents the easiest task with few distractors, Level 2 increases difficulty by adding more similar distractors, and Level 3 is the hardest with many closely resembling distractors and more complex layouts. The generalized analysis reveals that gaze stability (Fixation %) was broadly consistent across both cohorts. Romanian participants exhibited faster median reaction times than Portuguese participants; however, given the small sample size (N=7 per cohort), the presence of data-quality anomalies in three Portuguese sessions, and the absence of inferential statistical testing, this difference should be regarded as preliminary and hypothesis-generating rather than conclusive. By using standardized parameters, the results reduce accidental interactions and hardware noise, providing a reliable methodological basis for preliminary analysis and highlighting the need for larger sample sizes to establish statistical validity.

## Introduction

Eye tracking is a widely utilized technology for analyzing how individuals visually interact with digital environments through gaze measures^1–3^. By calculating where and for how long someone focuses on different areas of a screen, one can gain valuable insights into attention, decision-making processes, and cognitive load^4,5^. Recently, ET technology has seen significant application in serious game-based scenarios, where users dynamically engage with evolving visual representations^6^. Unlike static images, games elicit variation in visual attention during gameplay^6^, which can be quantified using gaze data collected by ET. Analyzing changes in gaze over time, along with the progression of games and interactions, is referred to as temporal analysis^7^. It can enable us to determine not only the areas to which people direct their gaze, which are often too static, but also approximate attention transitions throughout the gaming experience^8^.

Examining gaze behavior changes within a temporal framework along with fixation patterns, saccadic movements, and response times from interactive tasks in serious games offers deep insights that can enable (educators and psychologists do not manage this yet) to quantify attention deficits, evaluate the effectiveness of the intervention, and personalize learning experiences based on individual cognitive profiles^9,10^. Approximating visual attention is important both for identifying how quickly a player notices critical game elements or how their focus adapts to new challenges, which can inform individual attention, game development, and gameplay evaluation. Conventional gaze analysis often emphasizes aggregated data, such as heatmaps (fixation intensity and frequency), which may overlook vital time-sensitive dynamics. Adopting a chronological perspective in interpreting gaze measurements enables researchers to uncover patterns, such as hesitation, learning curves, or shifts in focal areas, which static summaries often fail to capture. This form of analysis is particularly beneficial in serious games or training simulations, where tracking attention flow could help to evaluate performance or engagement levels with greater precision and depth. To overcome the challenges above, this paper makes the following contributions:

- We propose a cognitive visualization tool named VisiTrail for time-series analysis tool that integrates multiple analytical dimensions: enhanced movement classification that distinguishes between eye-tracking metrics such as fixations, saccades, and smooth pursuit; temporal pattern analysis that reveals how attention evolves throughout task performance; object-click sequence tracking that directly links visual attention to user actions; and performance metrics that quantify both accuracy and efficiency of visual search behavior.
- We developed a standardized analysis pipeline to compare subject-specific variability in Portuguese and Romanian datasets. Two complementary threshold approaches are implemented: a subject-specific fixation threshold (721 px/s), derived from the 75th percentile of each participant’s gaze velocity distribution, and a generalized fixation threshold (483.70 px/s), representing the median of those percentiles across all sessions, to support both individual profiling and cross-cohort comparison. In addition, a reaction time window was defined using the 5th and 95th percentiles of the observed data distribution to exclude outliers, providing a uniform behavioral ’ruler’ that improves internal consistency and supports the replicability of comparisons across the two datasets.
- We provide a comprehensive tool that makes complex patterns, namely, the temporal dynamics of eye-tracking signals (fixations, saccades, and gaze transitions), their relationship to object-click sequences, and overall task performance (accuracy and efficiency) interpretable to researchers and practitioners, enabling them to develop personalized strategies aligned with users’ natural attention rhythms.
- The source code and demonstration video of VisiTrail are available (https://github.com/arnor-git/VisiTrail).

The rest of the paper is organized as follows. The section “Related work” discusses related work on eye tracking and learning environments. The section “VisiTrail (proposed tool)” presents our proposed approach for enhancing the Understanding of Time-Series Analysis of Eye-Tracking Data in interactive learning systems. The section “VisiTrail analysis and results” describes the experimental setup and results, including privacy and performance evaluation. Finally, the section “Conclusion and future work” concludes the paper and outlines possible directions for future research.

## Related work

Considering eye-based measures and relating them to human performance by identifying key indicators of attentional focus is common in many studies. For example, Frutos-Pascual et al.^11^ used ET to assess children’s behavior during attention-enhancement therapies. By analyzing eye movement patterns during interaction with puzzle games, the researchers found that participants with higher performance exhibited significantly different eye movement patterns than those with lower performance. Piazzalunga et al.^12^ used serious games-based ET data to identify children at risk of dysgraphia. By analyzing scan paths and fixation patterns during gameplay, the study found that children with poorer performance had chaotic scan paths. In contrast, those who performed better had more ordered scan paths. Accordingly, integrating ET metrics with game performance data provided a nuanced understanding of visual perception impairments. Velichkovsky et al.^13^ analyzed the distributions of fixation durations among professional, amateur, and novice eSports players. They found that highly skilled gamers exhibit greater variability in fixation durations and a bimodal distribution, indicating the presence of both ambient and focal fixation types.

Kim et al.^14^ aimed to develop a deep learning (DL) model to identify individuals with mental illnesses who have impaired visuospatial memory encoding. During a 3-minute memorization test of the Rey–Osterrieth Complex Figure Test (RCFT), eye movements were recorded to evaluate the structure and retention of psychosis, obsessive-compulsive disorder (OCD), and responses from healthy controls. The resulting scores and fixation points, which indicate areas of eye fixation, were used to train a Long Short-Term Memory (LSTM) model with an attention mechanism to distinguish between normal and impaired executive function. Argasinski et al.^15^ proposed a framework for developing and evaluating serious games that incorporate ET and biosensor data, suggesting that including temporal data is more effective in assessing user interactions and affective responses during gameplay. Hajari et al.^16^ explored team cognition by analyzing spatio-temporal ET data during laparoscopic simulation operations. Using Cross Recurrence Analysis (CRA) and overlap analysis, they identified features that distinguish high- and low-performing teams based on temporal patterns in gaze data, thereby enhancing understanding of collaborative performance.

Du et al.^17^ introduced PrivateGaze, a solution that protects users’ privacy while interacting with black-box gaze-tracking data, while maintaining estimation accuracy. PrivateGaze effectively preserves sensitive user data, such as identity and gender, as demonstrated through experiments conducted on four benchmark datasets. Paskovske et al.^18^ used ET to analyze how participants distributed their attention. Their findings revealed a notable difference between novices and experts based on the number of fixations. Experts tend to spend less time on tasks and employ more efficient problem-solving strategies. Since the prevalence of NDDs has been increasing globally, leading to a greater number of children with disabilities being integrated into mainstream schools, there is a call for equitable and appropriate support of learners with disabilities, driven by the implementation of educational policies and practices for inclusive education.

Previously, Costescu et al.^6^ primarily focused on developing a platform to enhance children’s play experiences. A key concept of this platform is identifying the focus of children’s attention during play activities, e.g., through the game “Mushroom Hunter,” which assesses the ability to sustain attention. This game was developed based on the go/nogo task, which is useful for assessing executive functions for continuous performance tasks^19^. Bueno et al.^20^ focused on developing an application specifically designed for AI research in education, particularly for children with NDD. Additionally, other studies^1,5^ explored how serious games built on design validated by psychologists and teachers, combined with eye-tracking technology, can provide insights to help teachers better support children with NDD. The study^21^ builds on research into the development of platforms designed to assist children with NDD. This is done by designing activities to practice executive functions and emotion regulation, supported by games with dedicated design that include elements accessible by sensory technologies. This study focuses on providing a time-series analysis tool that integrates multiple analytical dimensions. It includes enhanced movement classification that distinguishes among fixations, saccades, and smooth pursuit. The tool also offers temporal pattern analysis to reveal how attention evolves over the course of a continuous task. Additionally, it features object-click sequence tracking that directly links visual attention to user actions and reactions. Performance metrics quantify both the accuracy and efficiency of visual search behavior, making complex patterns interpretable to researchers and practitioners. This allows them to develop personalized strategies that align with the user’s natural attention rhythms.

While the current version of the proposed VisiTrail tool focuses on gaze-based time-series analysis, existing literature emphasizes that human attention is inherently multimodal. Auditory cues, in particular, significantly influence visual saliency and eye-tracking patterns, which are needed for accurate interpretation of visual attention. For example, Min et al.^22^ demonstrated that sound sources act as strong attractors for visual attention, especially in scenes with high audio-visual correspondence. Similarly, Min et al.^23^ proposed that incorporating audio saliency maps into traditional visual models significantly improves the accuracy of fixation prediction. Beyond saliency, the quality and synchronization of audio-visual signals also play a crucial role in user-perceived experience and attention allocation^24^. Recent advances in attention-guided neural networks have further demonstrated that late-stage fusion of auditory and visual features more closely resembles the temporal memory effects of the human perception system^25^. Although our current framework is optimized for the visual and motor constraints of neurodiverse populations in a digital game environment, future iterations of VisiTrail could incorporate auditory signal analysis to better account for cross-modal interaction, particularly in identifying whether a “distraction” was triggered by an on-screen visual distractor or an off-screen audio cue.

Moreover, the evaluation of user experience in digital educational tools is deeply rooted in perceptual quality assessment. As highlighted in comprehensive surveys by Zhai and Min^26^, the subjective experience of a digital interface influences the perceptual quality of the visual stimuli. In the context of serious games, the clarity and stability of the interface, often referred to as Screen Content Quality (SCQ), are critical for maintaining the attention of neurodiverse learners. Min et al.^27^ provide a benchmark for assessing screen content. They note that, unlike conventional natural scene content, screen content exhibits sharp edges and high-contrast text that require specialized quality modeling to optimize user engagement. To obtain overall Quality of Experience (QoE) in VisiTrail is assessed through the lens of perceptual video quality^28^, in which the temporal consistency of the “Mushroom Hunter” game affects participants’ cognitive load. By integrating these perceptual quality principles, VisiTrail moves beyond simple usability metrics toward a more rigorous quality-modeling approach. This can provide evidence that the recorded gaze patterns reflect the user’s cognitive state rather than being a response to poor signal quality or visual artifacts in the game environment.

The assessment of user engagement in digital environments is increasingly viewed through a multimodal lens. For the examined application, beyond purely visual stimuli, auditory cues have been shown to significantly shift saliency, particularly in scenarios with high audio-visual correspondence^22^. Furthermore, because the current study utilizes a digital interface, it is essential to consider the specific perceptual characteristics of screen-based media. Unlike natural scenes, screen content exhibits distinct statistical properties, such as sharp edges and high-contrast text, that require specialized quality benchmarks to ensure that the visual stimuli do not interfere with natural attention patterns^28^. A significant challenge in eye-tracking research is ensuring that recorded fixations reflect cognitive intent rather than reactions to hardware-induced distortions, preprocessing artifacts, or compression-related visual degradations. As demonstrated in unified blind quality assessment models, visual distortions in compressed natural or graphic content can fundamentally alter human gaze behavior^29^. Furthermore, Hessels et al. showed that spatial filtering and preprocessing choices in eye-trackers can substantially influence fixation detection outcomes, potentially biasing derived attention metrics^30^. By maintaining high-fidelity screen content in the “Mushroom Hunter” game and carefully validating fixation-threshold and reaction-time parameters, we aim to reduce the influence of visual and signal-processing artifacts, so that recorded gaze behavior more accurately reflects the attentional processes associated with NDD. In addition, the representativeness of the on-screen objects was ensured through a co-design process involving psychologists, educational researchers, and developers, who selected appropriate colors, object sizes, and object densities based on established attention-related characteristics observed in children with NDD^6^.

While some prior work has explored eye-tracking analysis in serious games and visual attention tasks^5,6^, existing tools typically focus either on high-precision laboratory analysis or on generic game analytics. Established platforms from eye-tracking developers, such as Tobii Pro Lab, provide optimized methods for controlled environments. In contrast, VisiTrail is specifically engineered for the “high-noise” context of inclusive education. Its core innovation is the *Generalized Parameter Validation* framework, which enables robust analysis of neurodiverse behavioral patterns using standard web-based hardware and user input.

## VisiTrail (proposed tool)

**Fig. 1:**
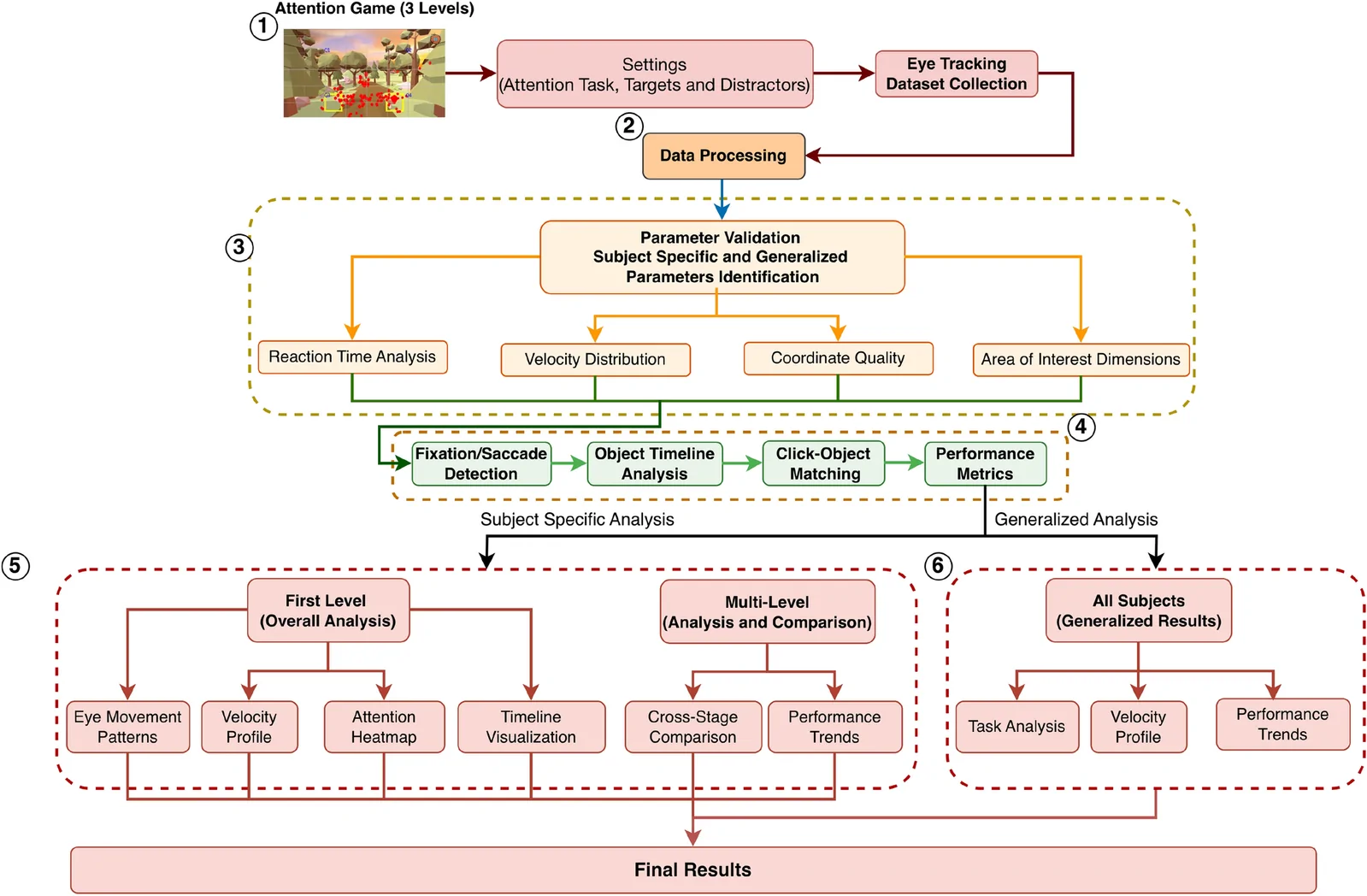
Proposed VisiTrail tool for Time-Series Analysis of Attention Game-based Eye-Tracking Data.

Figure 1 provides an overview of the proposed tool, covering data collection, processing, quality validation, parameter selection, gaze classification, and performance metrics for two scenarios: subject-specific analysis and general analysis across all subjects from the groups from Romania and Portugal. Subject-specific analysis yields two types of results: a first-level overall analysis that provides detailed insights and a multilevel analysis that compares performance across all three levels. Generalized analysis applies to all subject-based experiments.

Algorithm 1 implements a comprehensive eye-tracking analysis pipeline designed to enhance the understanding of time-series analysis of eye-tracking data for this context. The process begins by parsing coordinate strings from CSV files, then removing incomplete records and validating gaze positions within the screen boundaries, allowing a 50-pixel tolerance to account for eye-tracker imprecision, calibration drift, and minor head movements near the screen edges. Next, gaze behavior is classified using velocity-based thresholds. We select a threshold through subject-specific and generalized analyses to distinguish fixations, which indicate focused attention, from rapid eye movements associated with visual search behavior. The I-VT algorithm is then used to identify fixation events based on gaze velocity thresholds^31,32^. Following this, the algorithm employs greedy target-click matching with temporal constraints. A minimum reaction time helps prevent false matches from anticipatory responses, while a maximum time ensures that response windows remain realistic. The algorithm quantifies task performance using several metrics: the hit rate (indicating target detection accuracy), the false-alarm rate (reflecting attentional control), and the average reaction time (measuring processing speed). It also computes screen utilization and gaze path length to assess visual search strategies and patterns of attention distribution. Finally, the algorithm generates evidence-based recommendations for educators and psychologists based on validated performance thresholds derived from developmental and clinical guidelines.

**Algorithm 1:**
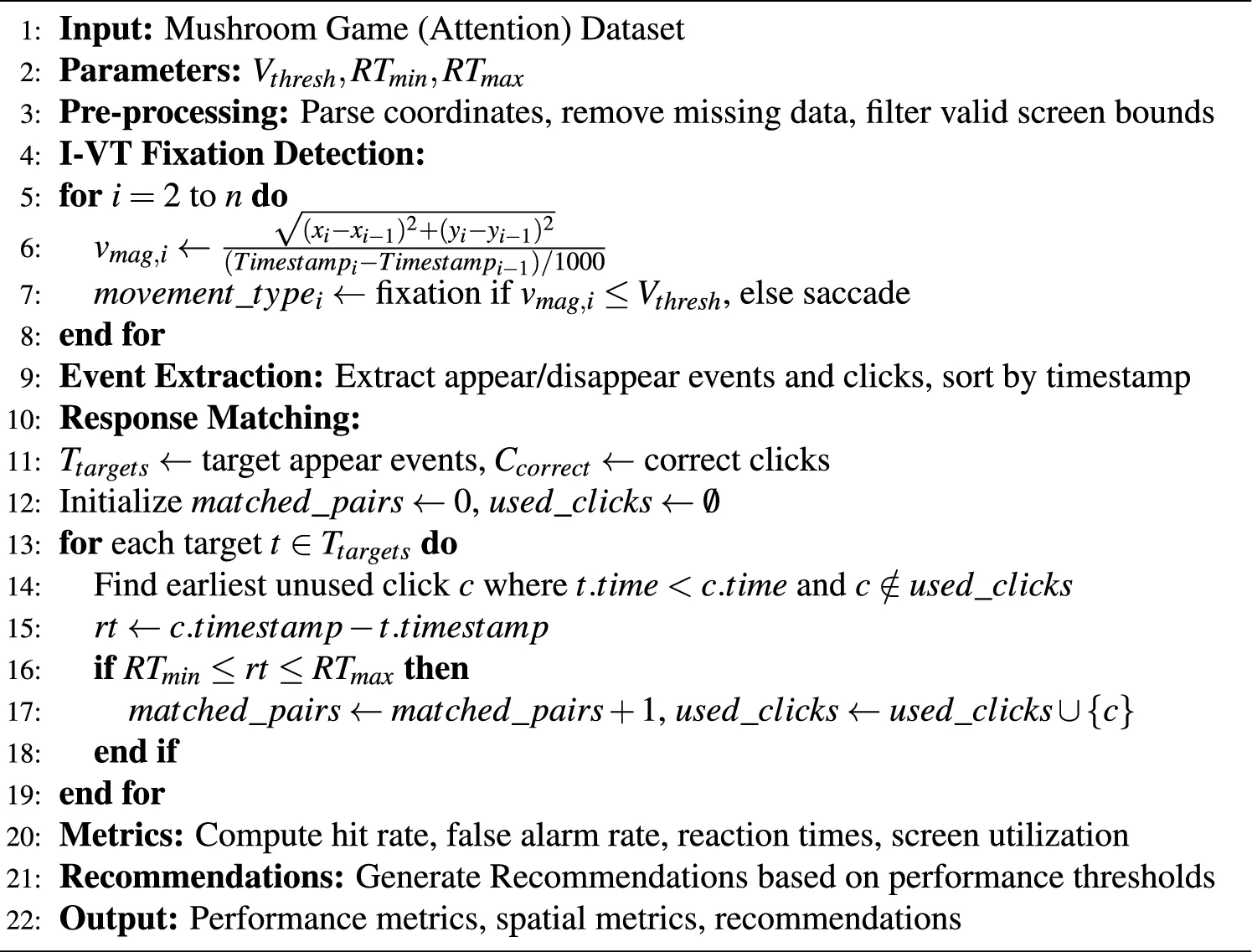
Algorithm for Time-Series Analysis of Attention Game-based Eye-Tracking Data (VisiTrail Tool)

### Dataset selection

Costescu et al.^6^ developed and structured the sustained-attention game used to collect data. It implements the Computerized Continuous Performance Task (CCPT) as originally described in^33^. Participants were shown a forest road with flowers (*distractors*) and mushrooms (*targets*) sporadically appearing on either side. The goal is to avoid distractions and to touch the screen when a target appears. Data was gathered using a Tobii Pro Nano eye-tracker (https://connect.tobii.com/s/article/how-to-configure-your-tobii-pro-nano-x2-or-x3-eye-tracker-with-the-mobile-device-stand?language=en_US), and the game utilizes a head-positioning and calibration screen specifically designed for the target demographic. While commercial eye-tracking solutions such as Tobii Pro Lab offer high-precision analysis for laboratory-grade hardware, they often lack the flexibility required for the high-noise environments typical of special education settings. The proposed framework addresses this gap by employing a generalized parameter-validation process (Fixation Threshold: 483.70 px/s; RT Window: 382.00-13564.15 ms), which represents the median of the 75th percentiles of gaze velocities across all sessions. This approach ensures that the tool remains resilient to the ocular-motor variability found in neurodiverse populations (ADHD, ASD, Dyslexia) across the Portugal and Romania datasets, providing a cost-effective and scalable alternative to traditional commercial software while maintaining the internal consistency required for comparative performance analysis. We use Pilot 3 data collected in Romania and Portugal. The Romanian dataset includes ET data from 8 students (1 student was removed due to missing 2-level data) at a special education school^6^. From Portugal, a dataset comprising 27 unique participants was collected. Among them, one participant completed only one stage (one file); one participant completed two stages (two files); seven participants completed exactly three stages (three files); and eighteen participants completed four or more stages of the game. For this study, only participants with exactly three files were included in the analysis, as these participants completed all three required stages of the game. Participants with fewer than three files were excluded due to incomplete data, whereas participants with more than three files were excluded to ensure consistency and comparability across subjects. Consequently, the final Portugal dataset used for analysis comprises seven participants, each contributing data from three game stages. Overall, we use data from 14 participants in the experiments. Furthermore, we consider this number sufficient to ensure diversity in participant behavior, especially since the study also incorporates data from participants in Romania, thereby increasing the variability and diversity of the overall dataset^6^.

### Pre-processing and data quality validation

The first step in the eye-tracking scoring system is to clean and prepare raw input data for difficulty levels 1, 2, and 3. The system begins by loading CSV files to extract eye position coordinates, which are initially stored as text strings (e.g., “(1250, 680)”). These strings are converted to numerical values for analysis, and object positions and area-of-interest (AOI) boundaries are extracted similarly. AOIs represent predefined screen regions associated with relevant game elements, such as target mushrooms or distractor flowers, enabling the analysis of how participants allocate visual attention during task performance. Next, the data are cleaned by removing invalid entries, such as records in which the eye tracker detected zero coordinates or where tracking was inaccurate. Incomplete records that could introduce errors into the analysis are also filtered out. The preprocessing phase adds necessary metadata, including student labels, difficulty levels, and normalized timestamps, to ensure proper sequencing and data integrity. Finally, the three-level files are combined into one comprehensive dataset. In the data quality validation step, we verified the accuracy of the ET data and removed erroneous entries. Eye trackers sometimes record implausible locations, such as negative coordinates or positions far outside the computer screen, so these values were excluded before analysis. Rows with missing ET data were also removed to ensure consistency and reliability in subsequent analyses. After preprocessing and validation, the cleaned dataset contains valid eye-tracking coordinates, game interaction events, and correctly labeled metadata, all of which are ready for detailed analysis.

### Parameter validation process

In this section, we discuss two types of settings for parameter settings based on subject-wise and generalized parameters.

#### Subject specific parameters and gaze classification

**Fig. 2:**
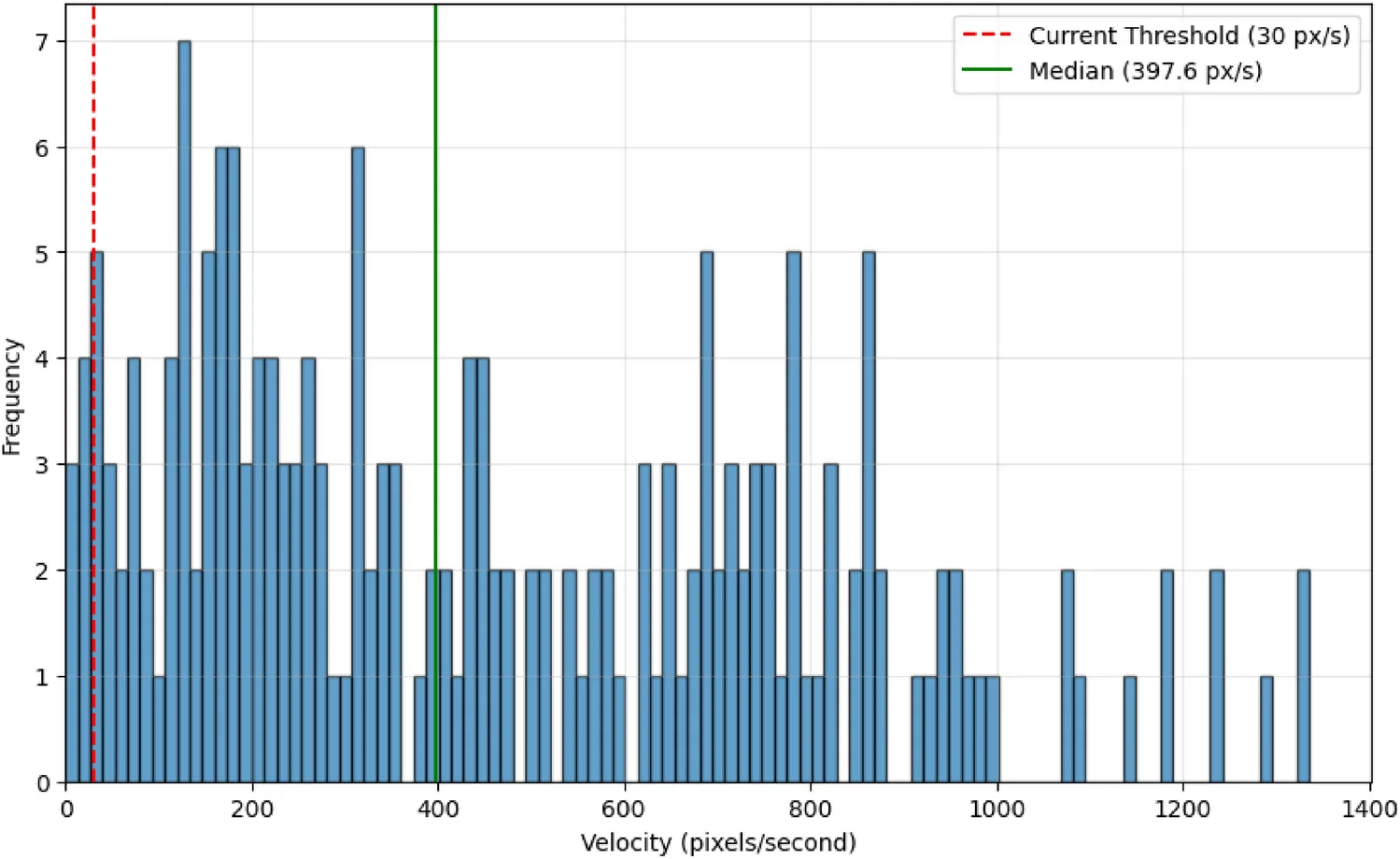
Subject Specific Distribution of gaze velocities (in pixels/second).

The parameter validation level underpins this ET analysis tool, employing a sophisticated, data-driven approach that fundamentally departs from conventional assumption-based methodologies used in cognitive assessment research. We focus on four levels: reaction-time analysis, velocity distribution, coordinate quality, and interaction area dimension. For reaction time, we measured the interval between object appearance and response by recording the object’s appearance time and the student’s response time. We found that most responses occurred between 522 milliseconds and 5000 milliseconds, so we used these as our minimum and maximum reaction times. Anything faster was likely an accident, and anything slower indicated that the student was not paying attention. Through velocity distribution analysis, we measured the distance between consecutive gaze points and divided it by the corresponding time interval to calculate gaze velocity^31,34^. We tested different velocity thresholds and found that 721 pixels per second provided the most effective cutoff. Lower values were classified as fixations, indicating relatively stable visual attention, whereas higher values were classified as saccades, representing rapid gaze shifts during visual search^35^. Our threshold selection was not arbitrary but based on systematic empirical validation of our eye-tracking data. The threshold for classifying fixations was set at 721 pixels per second, based on the 75th percentile of the gaze velocity distribution after removing outliers, as illustrated in Fig. 2. This threshold indicates that 75% of recorded gaze velocities fall below it, allowing us to conservatively identify slower movements as fixations while filtering out faster movements, which are likely saccades. The analysis revealed that commonly used lower thresholds, such as 30 pixels per second, captured only a small fraction of the data (approximately 4.2%) as fixations, significantly underestimating the actual number of fixations in this attention game dataset. Using the 75th percentile aligns the threshold with the data’s natural distribution, thereby improving the realism and accuracy of fixation detection in IVT analysis. The resulting velocity histogram and fixation rates support this choice, demonstrating that 721 pixels per second effectively distinguishes fixations from rapid eye movements in this specific dataset.

For coordinate quality analysis, we tested the strictness of ET accuracy by examining the number of data points that fell outside the screen boundaries. We then tested different tolerance levels to determine which retained the most accurate data while removing obvious errors^36^. The 50-pixel coordinate tolerance strikes a balance between data retention and tracking accuracy, preserving eye-tracking points while excluding clear measurement errors. Target success rates are computed by temporally matching target appearances to subsequent correct user clicks that occur within the predefined reaction time window. Spatial validation is handled separately through the game’s object-click interaction logic and area-of-interest definitions, ensuring that only clicks on the corresponding target objects are considered successful matches.

Each level used all validated parameters from the previous level to understand the student’s behavior throughout the gameplay. This level had four main steps: fixation/saccade detection^35^, object timeline analysis^37^, click-object matching, and performance metrics. First, we classified the students’ eye movements by examining their movement speed at each moment. Next, we created a timeline of all events that occurred during gameplay by reviewing the data and marking the instances when objects appeared on the screen, disappeared, or were clicked by the student. We then arranged these events in chronological order to visualize their sequence. After that, we matched up clicks with objects by looking at each time an object appeared and checking if the student clicked somewhere nearby within a reasonable amount of time; we used our validated settings (522-5000 milliseconds for timing) to decide if a click was meant for a specific object or if it was just random. Finally, we calculated performance metrics by counting the number of targets the student successfully clicked, the number of mistakes they made on distractors, their reaction speed, and the accuracy of their clicks, providing us with numbers that indicated how well the student was paying attention and responding during gameplay. After analyzing the data, we examined two scenarios: the first presented the overall analysis, providing a detailed breakdown of each student, and the multilevel analysis compared each student’s performance across all three levels. We create four different types of visual displays: (1) a timeline chart showing exactly when objects appeared and when clicks happened, (2) an eye movement pattern map showing where the student looked and how their eyes moved around the screen, (3) a velocity graph showing when their eyes were moving fast or slow over time, an attention heatmap with colored areas showing where they spent the most time looking plus markers for their clicks, and (4) a performance dashboard with charts showing their success rates and reaction times. For multilevel analysis, we created comparison charts that show how students’ performance changed from one level to the following, trend graphs that indicate whether they improved or worsened over time, and summary tables that display all the essential numbers side by side for easy comparison.

#### Generalized parameter and gaze classification

**Fig. 3:**
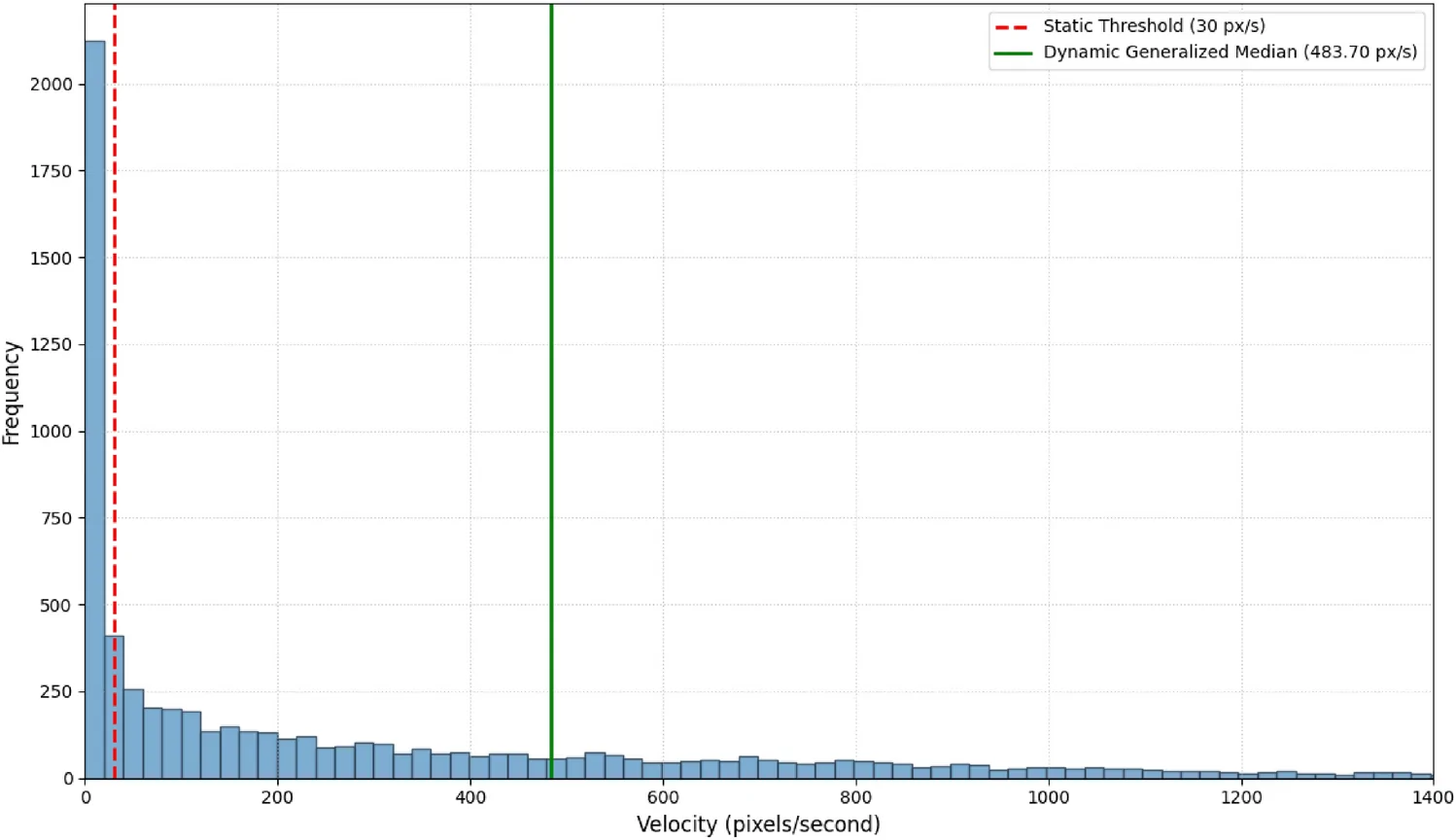
Generalized Distribution of Gaze Velocities (in pixels/second).

To ensure statistical rigor and facilitate a standardized comparison between the Portuguese and Romanian cohorts, we implement a generalized parameter validation process. While subject-wise thresholds were initially considered to account for individual calibration noise, a unified “behavioral ruler” was adopted to maximize internal validity. This approach ensures that performance differences are attributable to participant behavior rather than algorithmic sensitivity. The following parameters were dynamically derived from the aggregate pilot population (*N=14*) (see Fig. 3):

- **Gaze Velocity Threshold:** A generalized fixation threshold of 483.70 px/s was established, representing the median of the 75th percentiles of gaze velocities across all sessions. This threshold accounts for the hardware-specific noise floor inherent in web-based eye tracking. When visualized against the standard literature baseline of 30 px/s, the process effectively separates informational fixations from rapid saccadic movements.
- **Reaction Time (RT) Window:** To filter non-cognitive noise, the valid response window was defined using the 5th and 95th percentiles of all successful interactions across all subjects. The Minimum RT (382.00 ms) was set to exclude anticipatory or accidental clicks, while the Maximum RT (13564.15 ms) accommodates the visual search complexity of advanced levels. This ensures that delayed but intentional responses are accurately captured without being penalized as misses.

By replacing arbitrary constants with these evidence-based boundaries, the analysis provides a robust foundation for cross-cultural performance evaluation.

In this module, we present the results of the generalized gaze classification analysis, which evaluates the relationship between visual stability and task performance across the pilot population. The results are characterized by three primary metrics: Gaze Stability (Fixation %), Task Success (Hit Rate %), and Temporal Efficiency (Mean Reaction Time). Task success is determined by validated game interactions in which the user’s clicks correctly overlap with target object areas. At the same time, reaction times are computed by temporally matching the target’s appearance to the corresponding successful click event. By using a unified fixation threshold, we demonstrate a correlation between a participant’s ability to maintain a stable gaze and their overall accuracy in target identification. Furthermore, the reaction time density distributions highlight the cognitive processing rhythms of each cohort, providing a comparative visualization of behavioral patterns between the Portuguese and Romanian groups. These aggregated individual-file results validate the tracking environment’s consistency and the efficacy of the data-driven filtering parameters.

## VisiTrail analysis and results

This section demonstrates the results of the proposed tool for two scenarios: subject-specific and generalized.

### Subject specific results

**Fig. 4:**
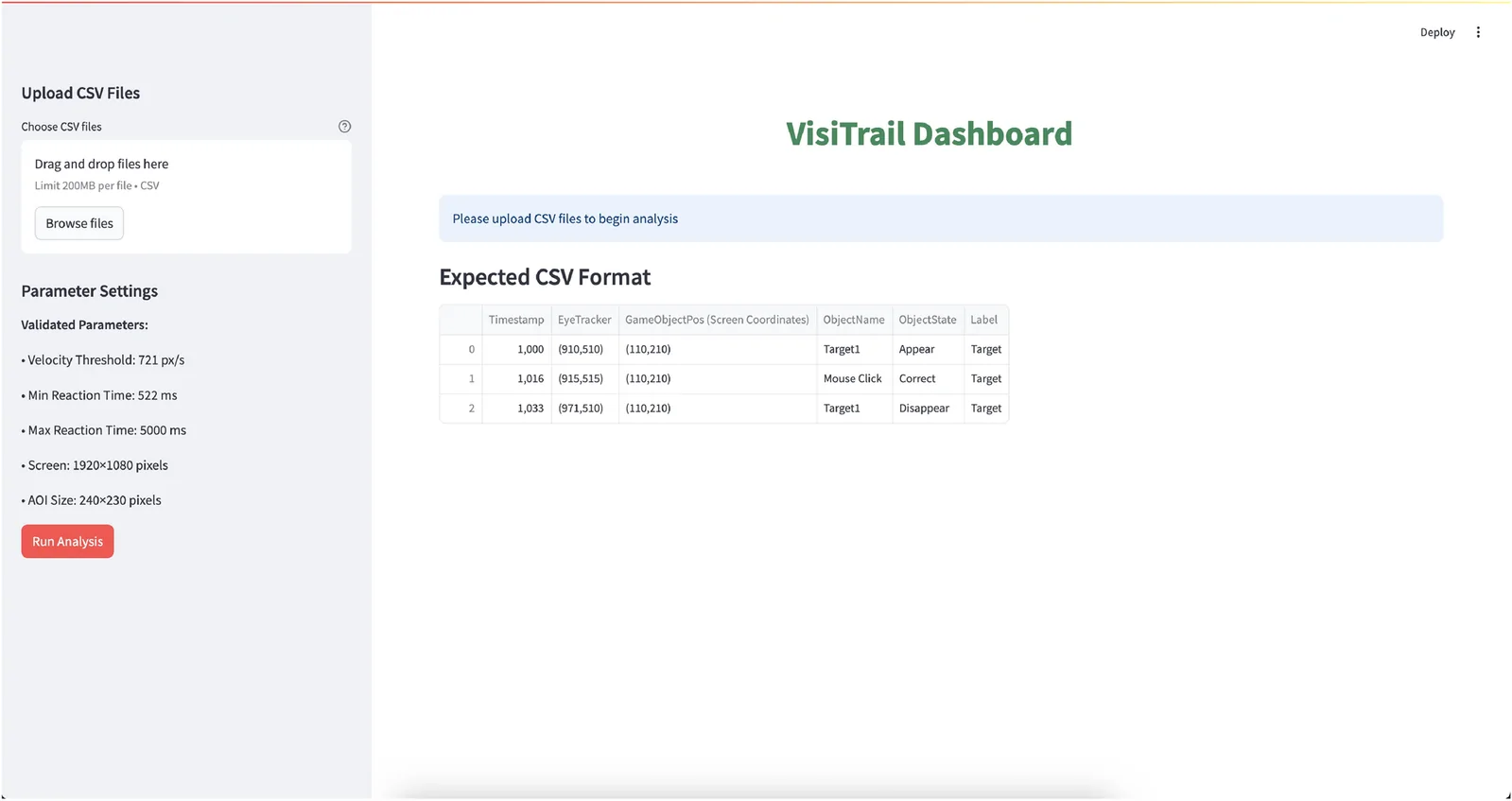
GUI of the VisiTrail (Sample 1).

**Fig. 5:**
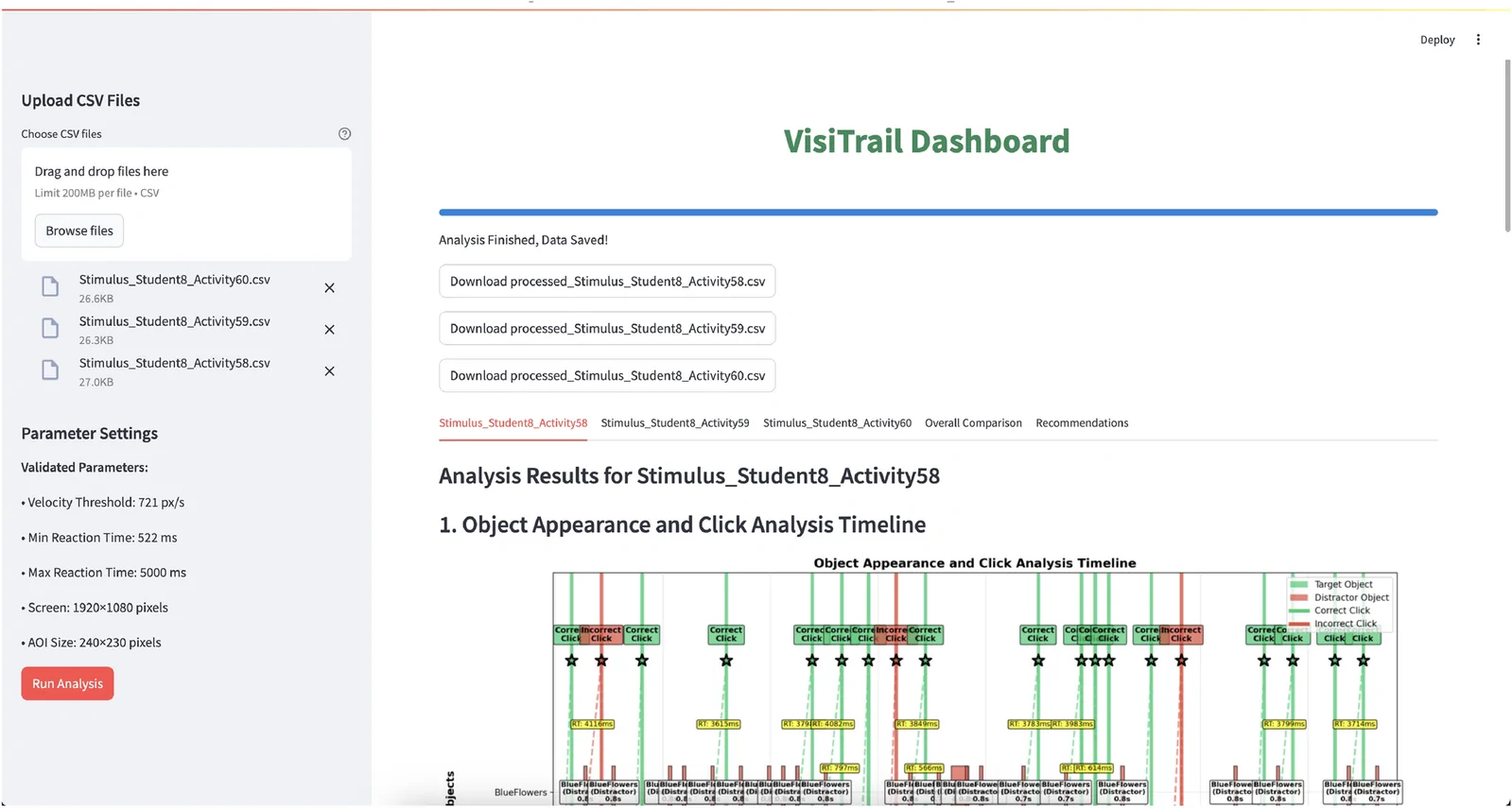
GUI of the VisiTrail (Sample 2).

Figures 4, 5 depict the Graphical User Interface (GUI) of the proposed VisiTrail Tool. The GUI initially displays the expected data format and predefined parameters. Further, it asks the user to input the attention game data. After entering the data, it generates 5 tables: the first three for individual game levels, the 4th for the overall comparison, and the 5th for recommendations. For this study, we focus on the data of a single student (Student 8) to gain insight into the student’s behavior.

**Fig. 6:**
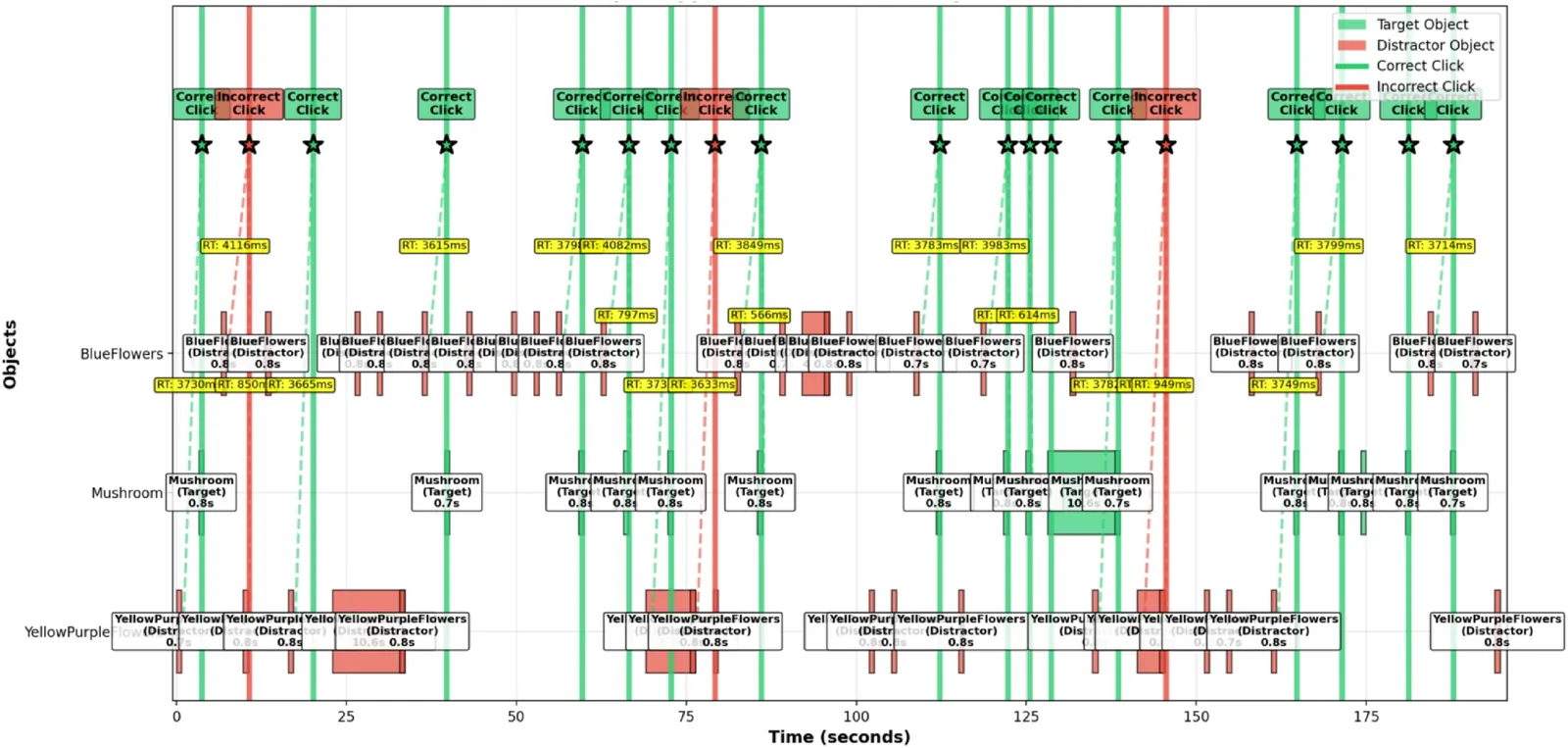
Temporal Analysis of Eye Tracking During Gameplay. The y-axis lists object types (targets and distractors), and the x-axis shows the task timeline. Green bars and lines indicate target appearances and correct clicks, while red/grey bars mark distractors. Red stars denote incorrect clicks on distractors.

Figure 6 provides a detailed chronological overview of a user’s performance in an attention game. The Y-axis categorizes the objects into three types: Mushrooms (the target), Blue Flowers, and Yellow-Purple Flowers (both distractors), while the X-axis represents the timeline of the task, spanning approximately 196.7 s at the first level of the gameplay. Each object’s appearance is marked by a horizontal bar, with green bars indicating the presence of target objects and red or grey bars representing distractors. Vertical green lines indicate when target objects appear, and green stars labeled “Correct Click” indicate when the participant successfully identified and clicked a target. In contrast, a single red star labeled “Incorrect Click” appears approximately 3 times, indicating the only instance in which the participant mistakenly clicked a distractor. During gameplay, the correct click is highlighted with a yellow box that displays the reaction time (RT) in milliseconds, indicating the time elapsed from target onset to the user’s response. Reaction times vary considerably, ranging from 566 milliseconds to 3900 milliseconds, suggesting variability in attention or response readiness. Over time, RTs improve, indicating potential adaptation or learning as the task progresses. Despite frequent, evenly spaced distractor presentations, the participant avoids nearly all of them, making only one incorrect click, demonstrating strong attentional control and task focus. In total, 16 target objects appear during the task, and the user correctly clicks on most of them, reflecting a very high level of accuracy. Each target object remains visible for about 0.8 s, during which the participant must respond quickly to register a hit. Distractors occur more frequently and last for similar durations, thereby increasing the task’s difficulty. A single mistake and a few slower reactions indicate occasional lapses, but these do not significantly affect the firm’s overall performance.

**Fig. 7:**
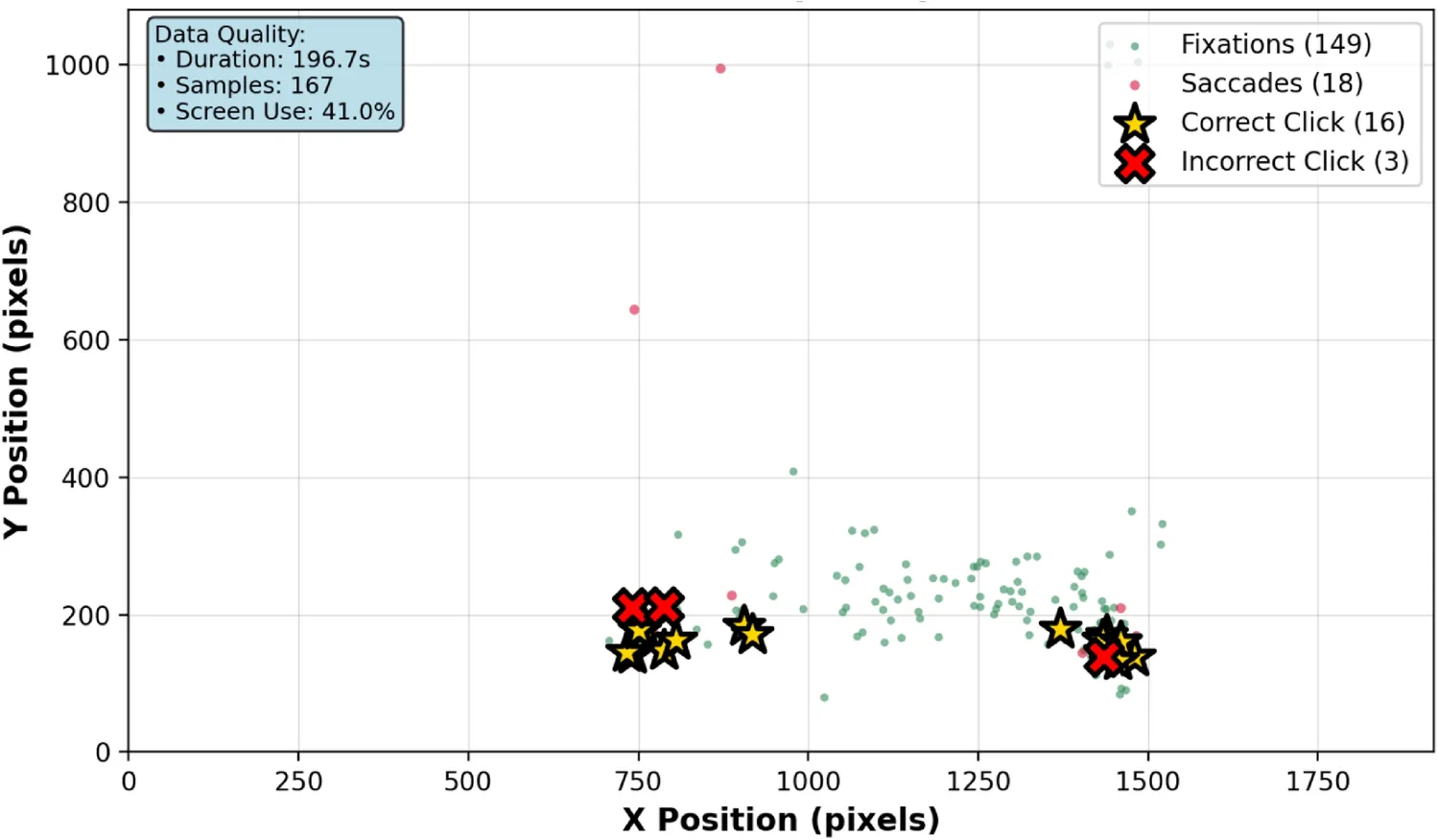
Eye Movement pattern Analysis. The pink dots indicate saccades (rapid eye movements between fixations), and the light green dots represent the students’ fixations. The yellow stars indicate correct clicks, and the red cross indicates incorrect clicks.

Figure 7 presents the eye movement patterns, indicating that students primarily looked at the AoIs. A total of 149 fixations were recorded, suggesting relatively active visual scanning behavior. Additionally, 33 Saccades were recorded, which typically represent short movements; their presence between fixation points indicates natural eye-movement dynamics. Large yellow stars indicate 16 correct clicks, suggesting that the user accurately identified and selected target elements on the screen. These correct clicks are organized into two main clusters, one on the left side and another on the right side of the screen, indicating where the targets were likely located. A single red X represents the only incorrect click, positioned near a group of correct clicks, which may indicate a near-miss error. The total duration of the task was 196.7 s, during which 167 gaze samples were recorded. The user engaged with only 4% of the screen area, suggesting that their focus was on specific regions rather than the entire interface. Overall, the user demonstrated high accuracy, selecting 15 of 16 targets, which corresponds to approximately 93.8%.

**Fig. 8:**
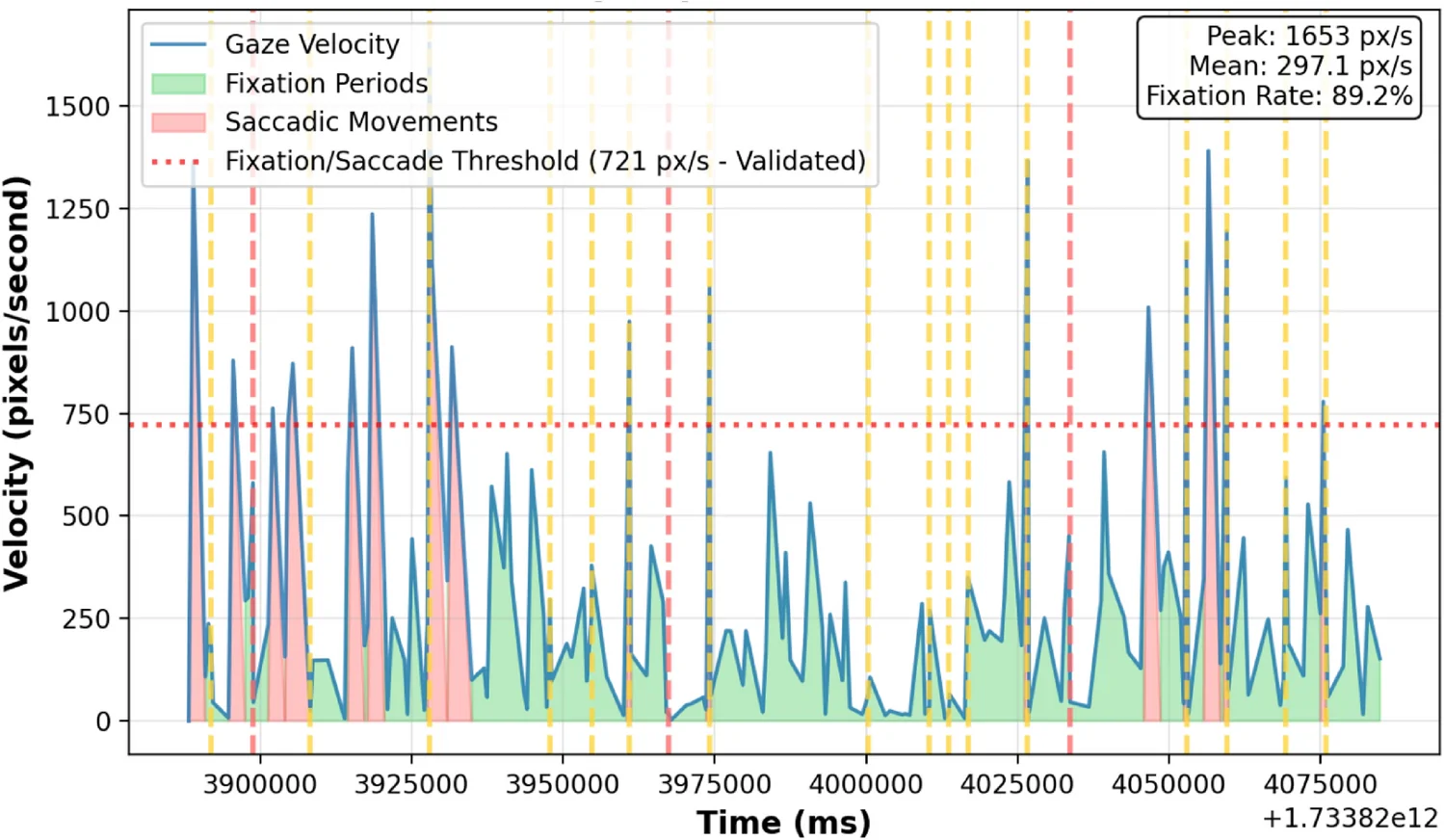
Eye Speed Over Time. The x-axis shows time (ms), and the y-axis shows gaze velocity (px/s). The blue line represents gaze velocity over time. Green areas indicate fixations (slow eye movement), red areas show saccades (rapid shifts), and the red dashed line marks the threshold distinguishing the two.

Figure 8 illustrates the gaze velocity of students during Level 1 gameplay. The x-axis represents time in milliseconds, while the y-axis shows velocity in pixels per second, reflecting how quickly a user’s gaze moves across the screen. The blue line tracks gaze velocity, capturing fluctuations in eye movement speed from moment to moment. Green-shaded areas indicate fixation periods, during which eye movement slows below a validated threshold, suggesting that the user is likely focusing on a specific area. In contrast, the red-shaded regions highlight saccadic movements, in which the eye rapidly shifts between points of interest. A red dashed line marks the fixation/saccade threshold at 721 pixels per second, distinguishing between slower fixation movements and faster saccades. Peaks in the velocity curve that exceed this line are interpreted as saccades. During the session, peak velocity reached 1643 pixels per second, whereas average velocity was 297.1 pixels per second. The fixation rate was 89.2%, indicating that most of the session was spent in visual fixation rather than scanning. Additionally, vertical dashed lines represent different click events, such as ’Correct’, ’Incorrect’, or ’Neutral’.

**Fig. 9:**
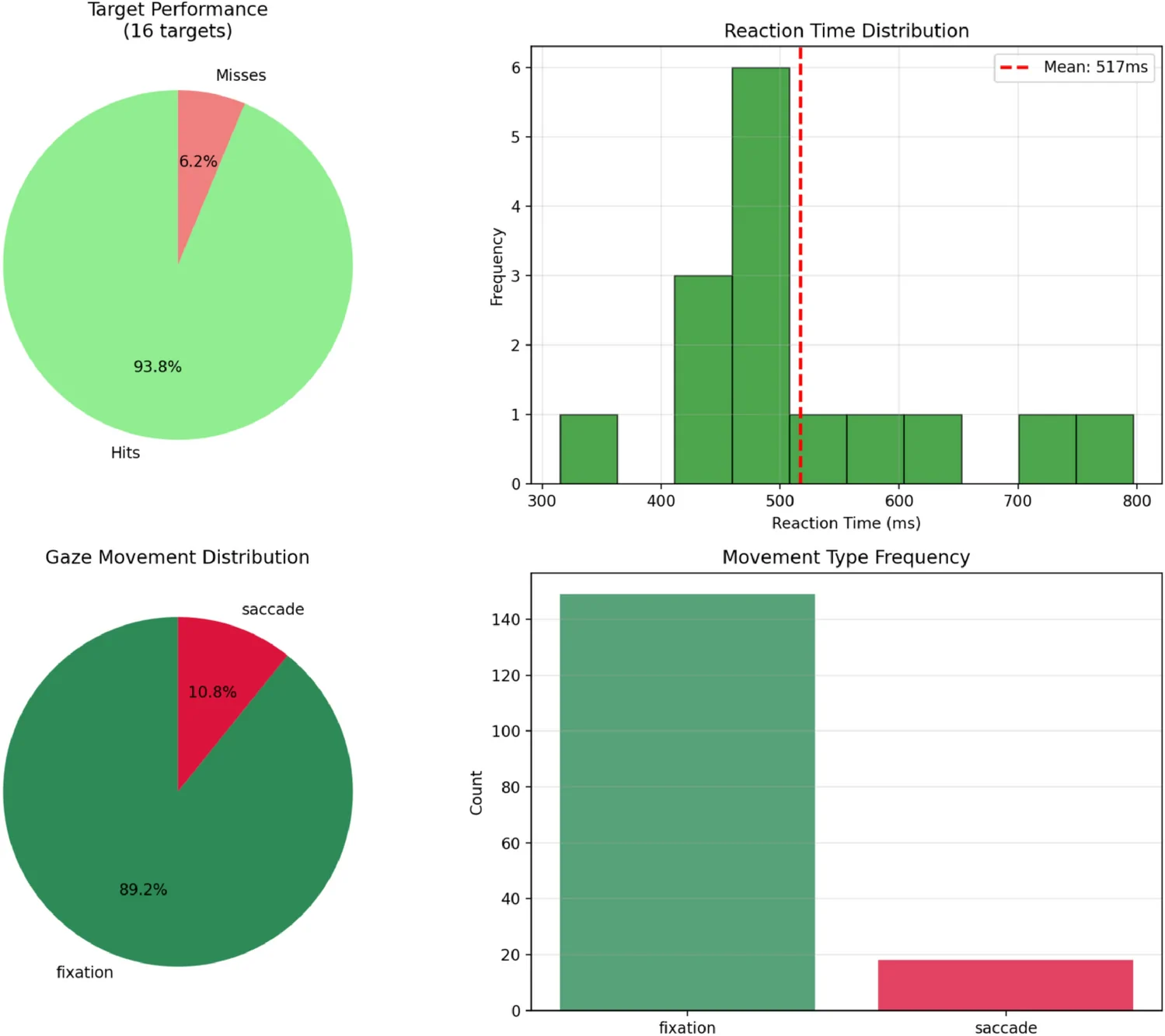
Performance Summary.

Figure 9 presents a comprehensive overview of students’ target-detection accuracy, reaction time, gaze movement, and movement frequency at level 1 of gameplay. In the top-left pie chart, the student demonstrated perfect accuracy, achieving 15 targets with 93.8% hits and 6.2% misses. The second graph (top-right bar graph) shows that most students’ reaction times fell between 400 and 550 ms, with a mean of 517 ms. This suggests consistent and relatively quick responses. The 3rd graph, bottom-left pie chart, reveals that 89.2% of the user’s eye movements were fixations, steady visual focus, while only 10.8% were saccades, which are rapid eye movements between points. Lastly, the fourth graph, bottom-right bar graph, visually reinforces this finding, showing that fixations occurred more than 140 times, whereas saccades were recorded only about 20 times. Together, these graphs indicate not only high task accuracy and efficient response times but also a gaze pattern dominated by stable visual attention.

**Fig. 10:**
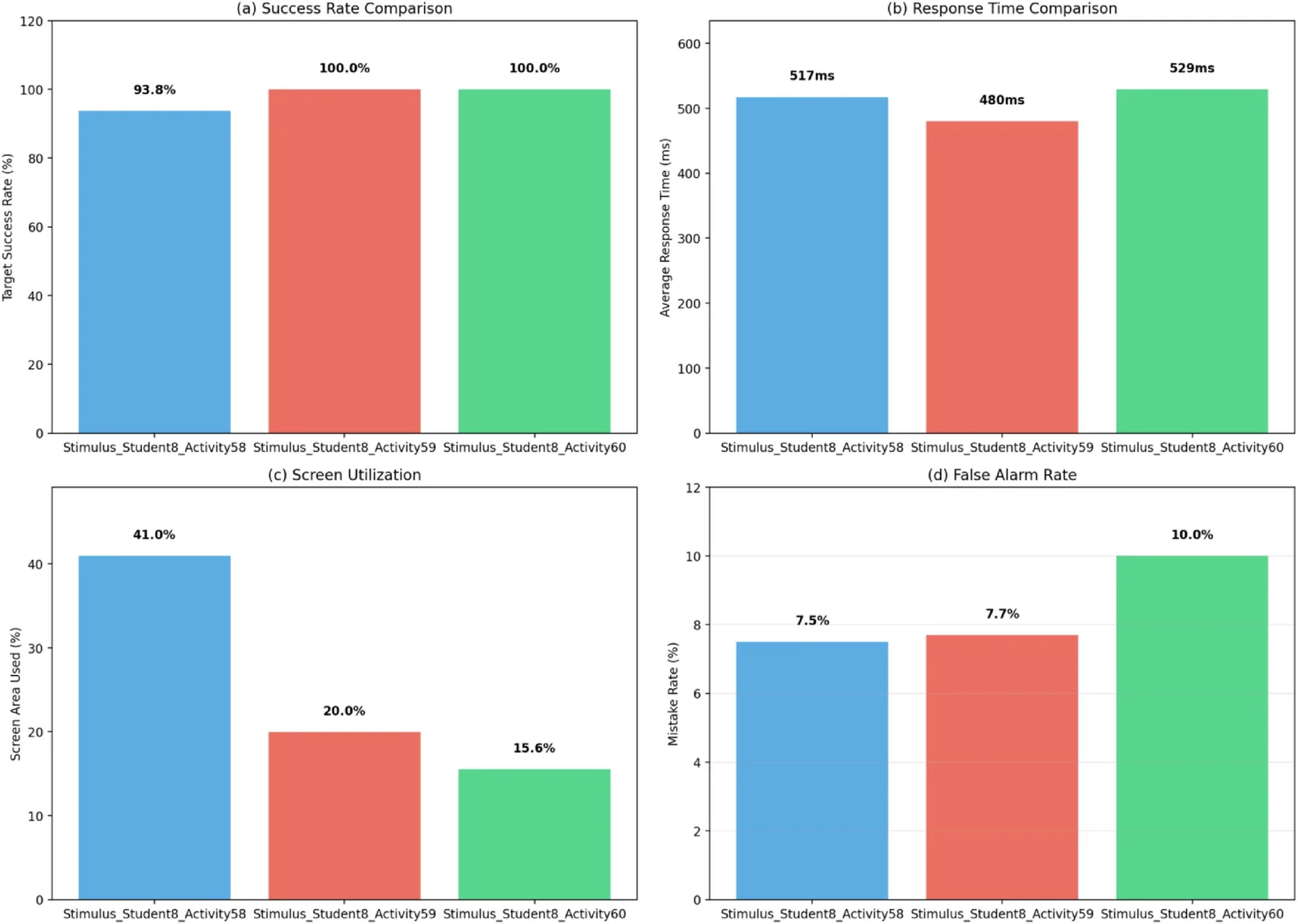
Multilevel Performance Comparison.

Figure 10 depicts 4 graphs. The first graph compares success rates; the second shows response times; the third displays total screen area used; and the fourth illustrates the number of errors. The highest target hit rate was achieved at the 2nd and 3rd level, whereas the lowest was at the 1st level. The highest time response time was on level 1, and the lowest was on level 2. Furthermore, the student occupies the most screen area at level 1 and is the weakest on level 3. Finally, the student made most of the mistakes in level 3.

The results reveal a clear picture of this student’s attention and learning patterns. In level 1, they struggled slightly, identifying approximately 94% of targets with a slow response time (517 ms), indicating that they were still learning the task. Levels 2 and 3 are the best performers, with a 100% success rate and significantly faster response times (480 ms at Level 2), demonstrating their ability to perform well when focused. However, level 3 showed a concerning increase in response time to 529 ms, suggesting that the participant may have become tired or overconfident, or that the task had become too complex. The student also spent less time on the screen over time (from 41% to 15.6%), indicating a high level of focus on the AoI. It can also be observed that the number of student errors increases with difficulty.

For psychologists, this data provides objective evidence of attention patterns that can be precisely observed, including when attention peaks and declines, how long the student can maintain focus, and whether interventions or medications are effective, as evidenced by comparisons of these metrics over time. Teachers can use this information to time their most important lessons during the student’s peak attention period (such as level 2), plan breaks before fatigue sets in (before level 3 declines), and recognize that this student requires extra support with impulse control, as their mistake rate increases dramatically when they are tired. Both professionals can work together, using these concrete data to develop personalized strategies that align with the student’s natural attention rhythms.

### Generalized results

**Fig. 11:**
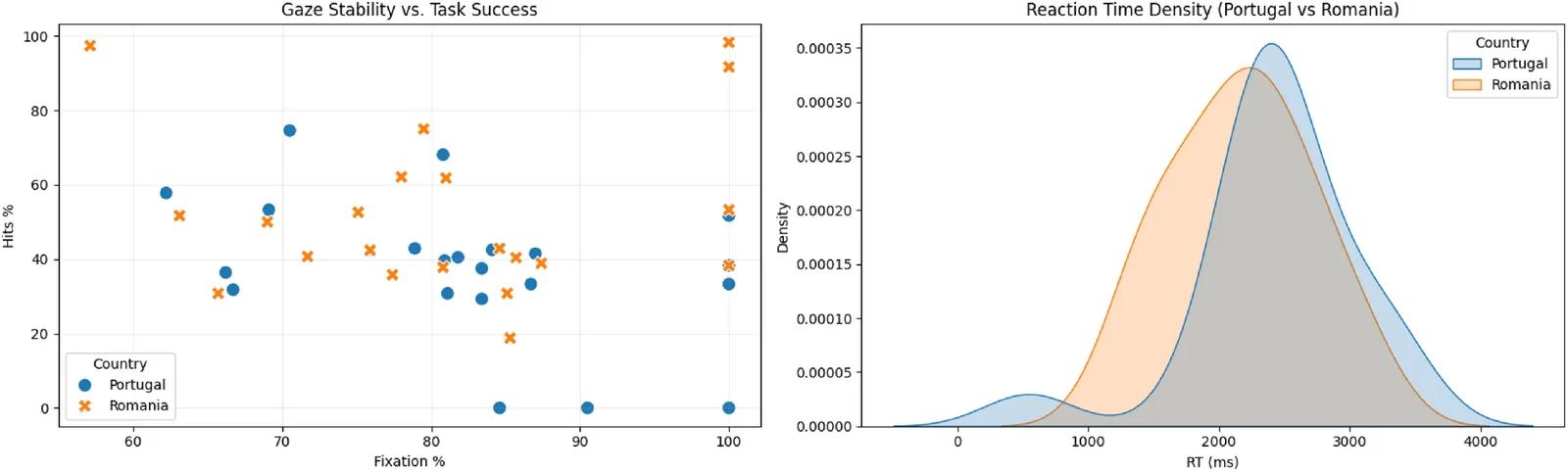
Generalizable relationship between gaze stability and task performance across populations. The positive correlation between fixation percentage and hit rate (left panel) was observed in both the Portuguese and Romanian samples, suggesting cross-national generalizability, despite differences in reaction-time distributions (right panel).

The left plot in Fig. 11 presents a scatter plot analysis examining the relationship between gaze fixation percentage and task performance (hit percentage) across participants from Portugal and Romania. The data reveal a generally positive correlation between gaze stability and task success, with most participants achieving fixation rates between 70% and 100% and corresponding hit rates between 30% and 75%. Notably, several outliers are present in the dataset, including participants with high fixation rates (approximately 100%) but minimal task success (near 0% hits), as well as instances of lower fixation (around 55%) associated with exceptionally high performance (approximately 98% hits). Both countries exhibit similar patterns in this relationship, suggesting that gaze stability is a meaningful predictor of task performance across these two populations. The second plot displays kernel density estimates of reaction-time distributions for Portuguese and Romanian participants. The analysis reveals a distinct difference in response latency between the two groups. Romanian participants exhibit a reaction time distribution with peak density centered around 2000-2500 ms, indicating relatively faster responses. In contrast, Portuguese participants exhibit a rightward-shifted distribution, with peak density near 3000 ms, suggesting slower average reaction times. Additionally, the Portuguese distribution appears broader, indicating greater variability in response times within this group. These findings suggest potential cross-national differences in cognitive processing speed or task approach strategies between the two participant samples.

Table 1 reveals significant variability in performance among Portuguese and Romanian pilot participants. Hit percentages range from 0% to 98.3% and reaction times span from under 600 ms to over 3,000 ms, reflecting considerable individual differences within and across cohorts. Fixation percentages are consistently high across participants, suggesting generally focused visual attention, but spatial screen coverage (usage %) exhibits anomalies exceeding 100% in several cases, likely attributable to repeated interactions with overlapping object areas rather than a measurement error. A positive relationship between response speed and task success is also observed, with faster reaction times tending to co-occur with higher hit rates. Prior to cross-cohort comparison, session files were screened for data integrity. Three Portuguese sessions were identified as tracking failures and excluded: one contained no recorded game objects whatsoever, while the remaining two recorded target objects but yielded zero successful responses, consistent with eye-tracker signal dropout rather than genuine task failure. Among the remaining 18 Portuguese and 21 Romanian valid sessions, Romanian participants achieved a higher mean hit rate (52.0%) than Portuguese participants (43.5%), with faster mean reaction times (2,167 ms vs. 2,435 ms). Given the small sample size and the absence of inferential statistical testing, these differences should be regarded as preliminary and hypothesis-generating rather than conclusive. These findings underscore the behavioral diversity of the participant sample and motivate larger-scale replication studies to establish statistical validity.

**Table 1:** Comparative Analysis of Ocular-Motor and Behavioral Biomarkers. The data reflect Gaze Stability (Fixation %), Spatial Screen Coverage (Usage %), and Target Success Rates for neurodiverse participants (*N=14*) from Portugal and Romania. Parameters were validated using a generalized fixation threshold (483.70 px/s) and a percentile-based response window (382.00-13564.15 ms).

Subject and Three Levels	Hits %	RT (ms)	Fix %	Usage %	Objects
pt_S11A74	0.0	0.0	84.6	26.4	0/3
pt_S11A75	33.3	548.0	86.7	49.2	1/3
pt_S11A76	0.0	0.0	90.5	53.5	0/4
pt_S15A241	30.8	3361.0	81.1	53.5	16/52
pt_S15A242	29.3	3105.0	83.4	49.6	17/58
pt_S15A243	42.5	2902.0	84.1	77.2	17/40
pt_S18A234	41.5	2486.0	87.0	76.1	17/41
pt_S18A354	42.9	2260.0	78.9	42.8	24/56
pt_S18A355	53.3	2361.0	69.1	68.0	32/60
pt_S25A294	36.4	2712.0	66.2	145.3	16/44
pt_S25A295	31.8	2399.0	66.7	195.4	14/44
pt_S25A296	57.8	2217.0	62.2	116.0	26/45
pt_S29A320	37.5	2375.0	83.4	69.9	21/56
pt_S29A321	74.6	2496.0	70.5	80.7	44/59
pt_S29A322	0.0	0.0	100.0	0.0	0/0
pt_S31A342	39.6	2174.0	80.9	80.3	19/48
pt_S31A343	40.5	1896.0	81.8	68.2	15/37
pt_S31A344	68.1	2525.0	80.8	72.0	32/47
pt_S4A68	33.3	3323.0	100.0	0.0	20/60
pt_S4A69	38.3	2330.0	100.0	0.0	23/60
pt_S4A70	51.7	2362.0	100.0	0.0	31/60
ro_Stimulus_Student10_Level156	52.6	2267.0	75.1	77.1	30/57
ro_Stimulus_Student10_Level157	30.8	1955.0	65.7	114.9	16/52
ro_Stimulus_Student10_Level158	50.0	2213.0	69.0	79.1	25/50
ro_Stimulus_Student11_Level177	38.9	2051.0	87.4	38.3	7/18
ro_Stimulus_Student11_Level178	75.0	1418.0	79.5	102.5	9/12
ro_Stimulus_Student11_Level179	18.8	1682.0	85.3	141.9	3/16
ro_Stimulus_Student12_Level175	53.3	2334.0	100.0	0.0	32/60
ro_Stimulus_Student12_Level82	38.3	2488.0	100.0	0.0	23/60
ro_Stimulus_Student12_Level84	37.8	1830.0	80.8	106.8	31/82
ro_Stimulus_Student2_Level4	97.4	1453.0	57.1	242.8	38/39
ro_Stimulus_Student2_Level44	91.7	1515.0	100.0	0.0	55/60
ro_Stimulus_Student2_Level45	98.3	1219.0	100.0	0.0	59/60
ro_Stimulus_Student6_Level24	35.8	2798.0	77.4	75.9	19/53
ro_Stimulus_Student6_Level25	40.7	1822.0	71.7	83.6	24/59
ro_Stimulus_Student6_Level26	51.7	2495.0	63.1	151.6	31/60
ro_Stimulus_Student8_Level58	40.4	3021.0	85.7	138.3	23/57
ro_Stimulus_Student8_Level59	42.9	2737.0	84.6	71.2	24/56
ro_Stimulus_Student8_Level60	61.8	2622.0	81.0	53.9	34/55
ro_Stimulus_Student9_Level64	30.8	3178.0	85.1	163.3	16/52
ro_Stimulus_Student9_Level65	42.4	2125.0	75.9	167.4	25/59
ro_Stimulus_Student9_Level66	62.1	2294.0	78.0	176.5	36/58

## Discussion and limitations

This section discusses key insights and limitations regarding VisiTrail’s impact on eye-tracking data analysis. While VisiTrail enhances the understanding of gaze behavior in relation to task performance, individual differences among participants may influence the results. The tool also reveals important trends across diverse cultural groups, but its reliance on standardized parameters may overlook contextual factors that affect gaze dynamics. Future research could address these limitations by incorporating more diverse participant samples and exploring additional influences on visual attention and task performance in interactive settings. The shift from subject-specific to generalized parameters has led to a more conservative performance profile, as evidenced by a decrease in participant success rates. This change is largely due to the standardized reaction time window and dynamic fixation threshold, which filtered out faster, potentially accidental interactions, and offered a more realistic assessment of intentional target identification. Ultimately, the generalized approach establishes a more rigorous “behavioral baseline,” ensuring that cross-cultural comparisons between Portugal and Romania are not inflated by individual hardware noise or local data artifacts. The current analysis uses an expanded dataset (*N=14* students; *N=42* sessions) to establish a more robust psychometric baseline, mitigating the risk of parameter over-tuning to individual sessions by deriving thresholds from the aggregate population distribution. This ensures that the analysis framework generalizes across participants rather than reflecting session-specific noise. This standardized framework (Fixation Threshold: 483.70 px/s; RT Window: 382.00-13564.15 ms) provides the statistical power necessary for meaningful cross-cultural comparisons.

## Conclusion and future work

This paper presents an eye-tracking analysis tool that examines time-series data from eye-tracking during task performance and gaze measurements. It includes an analysis of how attention changes over time, tracks the sequence of clicks on objects to connect visual attention with user actions, and provides metrics to measure both accuracy and efficiency. The tool is designed for two main scenarios: analyzing individual participants and comparing results across all participants from Romania and Portugal. In the subject-specific analysis, data from a single participant are used to generate two types of outcomes: a comprehensive overall analysis that provides in-depth insights and a multilevel analysis that compares performance across three levels. The generalized analysis, which includes data from all subjects, shows that although gaze stability was similar between the groups, the Romanian participants exhibited greater variability in reaction times than the Portuguese participants. By using standardized parameters, the findings effectively filtered out random hardware interactions and noise, providing a reliable basis for comparing visual attention and task performance across cultures.

In the future, we plan to design Large Language Models (LLMs) to enhance this eye-tracking tool by making it more intelligent and user-friendly (e.g., LLMs can automatically label gaze patterns, generate easy-to-understand summaries of user behavior, and enable researchers to interact with the tool via simple language commands). This means users could ask questions like “When was the user most distracted?” and get helpful insights without needing complex code or manual analysis.)

## Data Availability

The datasets generated and/or analysed during the current study are available from the corresponding author on reasonable request.
